# Intralymphatic mRNA vaccine induces CD8 T-cell responses that inhibit the growth of mucosally located tumours

**DOI:** 10.1038/srep22509

**Published:** 2016-03-02

**Authors:** Lukasz Bialkowski, Alexia van Weijnen, Kevin Van der Jeught, Dries Renmans, Lidia Daszkiewicz, Carlo Heirman, Geert Stangé, Karine Breckpot, Joeri L. Aerts, Kris Thielemans

**Affiliations:** 1 Laboratory of Molecular and Cellular Therapy, Vrije Universiteit Brussel, Laarbeeklaan 103E, 1090 Brussels, Belgium; 2 Diabetes Research Center, Vrije Universiteit Brussel, Laarbeeklaan 103E, 1090 Brussels, Belgium

## Abstract

The lack of appropriate mouse models is likely one of the reasons of a limited translational success rate of therapeutic vaccines against cervical cancer, as rapidly growing ectopic tumours are commonly used for preclinical studies. In this work, we demonstrate that the tumour microenvironment of TC-1 tumours differs significantly depending on the anatomical location of tumour lesions (*i.e.* subcutaneously, in the lungs and in the genital tract). Our data demonstrate that E7-TriMix mRNA vaccine-induced CD8^+^ T lymphocytes migrate into the tumour nest and control tumour growth, although they do not express mucosa-associated markers such as CD103 or CD49a. We additionally show that despite the presence of the antigen-specific T cells in the tumour lesions, the therapeutic outcomes in the genital tract model remain limited. Here, we report that such a hostile tumour microenvironment can be reversed by cisplatin treatment, leading to a complete regression of clinically relevant tumours when combined with mRNA immunization. We thereby demonstrate the necessity of utilizing clinically relevant models for preclinical evaluation of anticancer therapies and the importance of a simultaneous combination of anticancer immune response induction with targeting of tumour environment.

Cervical cancer has become the fourth most common neoplastic disease affecting women and the fourth leading cause of women’s death worldwide, now exceeding 500,000 new cases every year^1^. Since the discovery of the link between cervical cancer and Human Papilloma Virus (HPV)-infection, it has been shown that this relationship is stronger than the correlation between smoking and lung cancer or the one between liver cancer and Hepatitis B Virus-infection^2^,^3^. In recent decades, extensive research efforts have led to the development of potent prophylactic anti-HPV vaccines. Currently, two vaccines are commercially available and several others are under investigation^4^. However, the vaccine coverage is still not optimal in many countries. In addition, although some evidence of cross-protection against other HPV subtypes (*i.e.* those that are not included in the vaccine) has been reported, no therapeutic potential of the two vaccines has been demonstrated so far, most likely due to the fact that the targeted molecules disappear when cancer becomes established^5^. The two most common HPV strains endowed with oncogenic capacity are HPV16 and HPV18 (estimated as a primary cause of 70% of invasive cervical carcinomas)^6^. Their genome encodes two oncoproteins of special interest: E6 and E7, which in case of persistent infection, contribute to the raise and maintenance of the malignant phenotype. Expression of these oncoproteins is one of the major hallmarks of HPV-related tumours, making cervical cancer a particularly appealing model for immunotherapy. In this regard, many different therapeutic approaches have been evaluated so far, including protein- and peptide-, DNA-, viral and bacterial vector- and cell-based vaccines^7^,^8^. However, despite many advances and promising preclinical data, the clinical efficacy of therapeutic vaccines demands further improvement, especially for the patients with advanced cervical cancer, now considered mainly for palliative treatment^9^.

As the average success rate of translation of preclinically tested anticancer treatments to clinical trials is less than 8%, it has been suggested that therapeutic failure of some clinical trials might be associated with an overestimated potency of the vaccines at the preclinical level^10^,^11^. Additionally, a growing body of evidence highlights the complexity of T-cell homing pathways to mucosally located tumour lesions. It has therefore been postulated that the mucosal immunization route predetermines the mucosal homing programme of induced CD8^+^ T cells, thus allowing them to access mucosally located tumour lesions^12^,^13^. In the same vein, it is being hypothesized that the subcutaneously implanted tumours are not optimal as models for preclinical evaluation of therapies targeting HPV-related tumours^14^,^15^. Although orthotopic tumour models were shown to be more predictive of treatment responses than subcutaneous tumours, only a few research groups took the effort to implement more rigorous criteria for preclinical therapy evaluation in animal models^16^,^17^. It is therefore critical to improve the translational value of animal experiments by utilizing models that more accurately reflect the human pathology and allow for better assessment of new therapies^11^,^18^.

The field of messenger ribonucleic acid (mRNA)-based therapeutics has undergone a rapid progress resulting in the transition from an extensive preclinical testing to phase III clinical trials for anticancer vaccines^19^. Based on the experience our group has gained in this field over the last decade, we designed an mRNA-based vaccine encoding for immune-stimulating signals: CD40 Ligand (CD40L), constitutively active Toll-like receptor 4 (caTLR4), and CD70, collectively called TriMix. This mRNA-vaccine was administered into the subiliac lymph nodes of C57BL/6 mice and showed curative potential in a number of subcutaneously implanted tumours^20^.

In this study, we employed clinically relevant laboratory models of HPV-related tumours to evaluate the TriMix vaccine administered together with mRNA encoding the HPV16-E7 oncoprotein. The therapeutic efficacy of our approach was assessed separately for TC-1 tumour lesions located at different anatomical sites: subcutaneously, in the lungs and in the genital tract, providing evidence of a tremendous impact of tumour lesion location on the outcome of the tested therapy. We demonstrated that the vaccine-induced CD8 T cells migrate to the tumour tissue and suppress tumour growth despite the fact that they do not express the markers associated with mucosal location. Moreover, we evaluated the role of tumour microenvironment (TME) on the therapeutic outcome for each of the tested tumour locations. In the context of genital tract tumours, we demonstrated that the immunosuppressive TME could be attenuated by chemotherapy with cisplatin, thus significantly decreasing the numbers of myeloid-derived suppressor cells (MDSCs) and regulatory T cells (Tregs). Combination of cisplatin treatment and E7-TriMix immunotherapy rendered the mice tumour-free.

## Results

### T cells induced by E7-TriMix mRNA migrate specifically to tumour lesions

**Figure 1:**
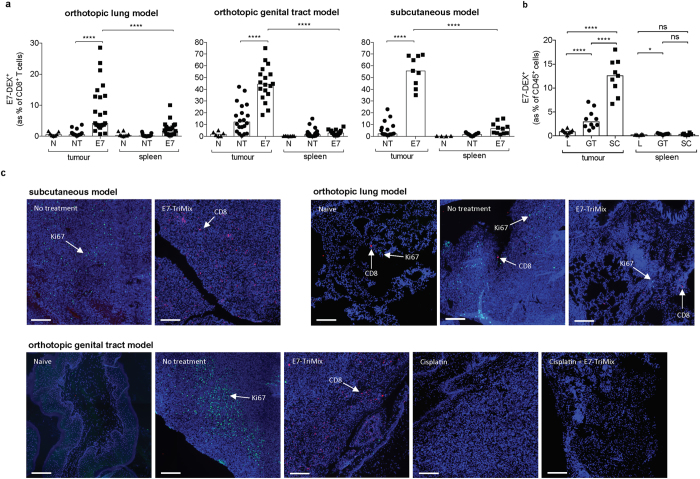
E7-TriMix mRNA immunization induces high numbers of E7-specific T cells that differentially migrate into the tumour tissue depending on its location. Mice bearing differently located fluc^+^ TC-1 tumours were immunized by intranodal injection with E7-TriMix and tumour tissue and spleens were isolated for immune response monitoring as described in Materials and Methods. Flow cytometric analysis was performed to evaluate the numbers of E7-specific CD8^+^ T cells, represented as a percentage of CD8^+^ T cells (**a**) or percentage of CD45^+^ cells (**b**). Each dot represents an individual mouse; the bars correspond to the median values. Immunofluorescence analysis was performed on frozen tumour sections to detect Ki67^+^ cells (green) and CD8^+^ T cells (red) in lung tissue, genital tract and subcutaneous tumours (**c**). Nuclei were stained with DAPI (blue). The scale bars correspond to 200 μm. Abbreviations: N: naïve, NT: no treatment, E7: E7-TriMix-treated mice, L: lungs, GT: genital tract, SC: subcutaneous tumours. (**a**,**b**) data shown from 2–4 independent experiments, 4–28 individuals per group. (**c**) representative photos from 2 experiments, 2 mice per group. Statistical analysis: Mann-Whitney test; * significant at p < 0.05, **** significant at p < 0.0001, ns: non significant.

Mice bearing subcutaneous, lung or genital tract TC-1 tumours were treated according to the experimental setting described in Materials and Methods. Ten days after the last immunization, a randomized group of animals was sacrificed to evaluate the antitumour immune responses. To this aim, the immune cells were isolated from the tumour tissue and spleens. As shown in Fig. 1a, E7-dextramer staining revealed that the vaccine-induced CTLs (E7-DEX^+^ CD8^+^ T cells) were endowed with the capacity to migrate to the tumour nest, constituting approximately 10% to 50% of tumour-infiltrating CD8^+^ T cells, depending on the tumour location. In this respect, the fraction of E7-specific T cells was most elevated for the subcutaneous model. Importantly, the percentage of E7-specific T cells within the total tumour immune infiltrate (defined as the CD45^+^ population) was approximately 39-fold higher in the tumour nest as compared to spleen in the subcutaneously implanted tumours, and 7- and 3-fold higher in genital tract and lung lesions, respectively (Fig. 1b). Tumour infiltration by CD8^+^ T cells was also confirmed by immunofluorescent staining of frozen tumour tissues (Fig. 1c). A striking observation is that a higher CD8^+^ T-cell infiltration is accompanied by a lower number of proliferating cells (Ki67^+^).

### Tumour-infiltrating E7-specific T cells express PD-1 but maintain the capacity to secrete IFN-γ

**Figure 2:**
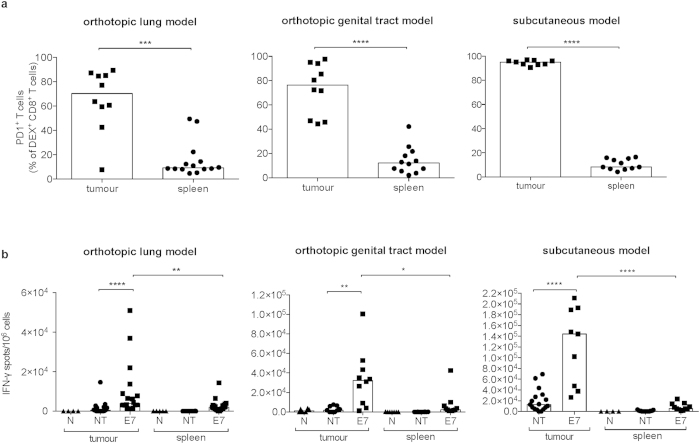
Tumour-infiltrating E7-specific T cells express PD-1 and secrete INF-γ in response to antigen stimulation. Fluc^+^ TC-1 tumour-bearing mice were immunized with E7-TriMix mRNA and tumour/spleen tissue was excised as described in Materials and Methods. Freshly isolated splenocytes or tumour-infiltrating immune cells were checked for the expression of PD-1 (**a**). An *ex vivo* IFN-γ ELISPOT assay was performed on tumour-infiltrating immune cells and sorted CD8^+^ spleen cells (**b**). The values shown for (**b**) were normalized based on the numbers of CD8^+^ T cells determined by flow cytometry. The background obtained for cells not stimulated with E7 peptides was subtracted. Each dot represents an individual mouse; the bars correspond to the median values. Abbreviations: N: naïve, NT: no treatment, E7: E7-TriMix-treated mice. (**a**) 2 independent experiments, 9–14 individuals per group; (**b**) 2–3 independent experiments, 4–20 individuals per group. Statistical analysis: Mann-Whitney test; * significant at p < 0.05, ** significant at p < 0.01, *** significant at p < 0.001, **** significant at p < 0.0001.

We further evaluated the phenotype of E7-DEX^+^ CD8^+^ T cells and found that they expressed high levels of Programmed Cell Death Protein 1 (PD-1) (Fig. 2a). The well-documented T-cell exhaustion paradigm prompted us to investigate whether these cells were still functional and antigen-responsive. To this end, an IFN-γ ELISPOT assay was performed and provided evidence that the tumour-derived T cells secrete IFN-γ upon incubation with synthetic E7 peptides. This phenomenon was particularly pronounced for the subcutaneous tumour model (Fig. 2b). Interestingly, a similar pattern as for the E7-dextramer staining was observed for IFN-γ secretion, *i.e.* the numbers of spot forming units (SFUs) were higher for cells isolated from the tumour tissue than from the spleen (more than 16-fold higher in the subcutaneous tumours, 13-fold higher in genital tract and almost 3-fold higher in lung lesions).

### Tumour-infiltrating T cells do not express markers associated with mucosal location

**Figure 3:**
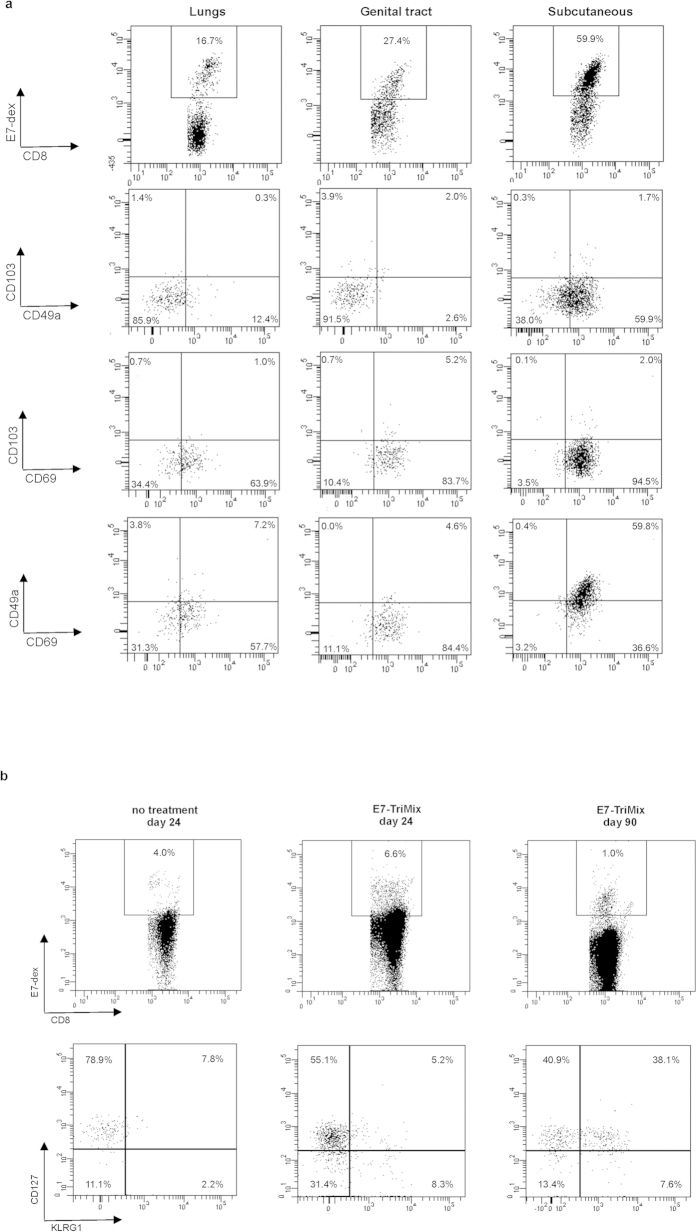
Tumour-infiltrating E7-specific T cells do not express mucosa-associated markers and their splenic counterparts display the phenotype of central memory cells. Mice bearing differently located fluc^+^ TC-1 tumours were immunized with E7-TriMix and tumour tissue and spleens were isolated 10 days after the last immunization as described in Materials and Methods. Flow cytometric analysis was performed to assess the expression level of mucosa-associated markers: CD103, CD49a and CD69. Survivors that had rejected genital tract tumours were killed 90 days after the last immunization and memory phenotype was evaluated based on the expression pattern of CD62L, CD127 and KLRG1 on E7-DEX^+^ cells. Dot plots representative for 6 individuals per group are shown for mucosal markers (**a**) and memory profile (**b**).

A growing body of evidence points to the existence of the so-called tissue resident memory cells (Trm)—a T cell population that permanently resides in peripheral tissues and does not enter the circulation. Therefore, in the context of mucosally located tumours it has been postulated that T cells could be generated in the process of imprinting, in which they are primed by dendritic cells derived from a mucosal tissue and thus endowed with the capacity to penetrate into and retain within this tissue^13^,^21^,^22^. Additionally, several convincing reports about the beneficial impact of Trm cells in the context of both infectious diseases and cancer exist^23^,^24^. Hence, it is being postulated that Trm cells should become a new criterion for determining the successful development of therapeutic anticancer vaccines^25^. For that reason, in our current work we decided to use the mucosal T cell markers that—according to current knowledge—are indicative of the Trm cell phenotype. Therefore, we checked the E7-DEX^+^ CD8^+^ T cells for the expression of CD103, CD49a and CD69^22^,^26^. We found that neither CD103 nor CD49a were expressed within the population of tumour-infiltrating E7-specific T cells in the orthotopic models. In contrast, almost all E7-DEX^+^ CD8^+^ T cells were CD69 positive in all tested tumour locations, with a large fraction of CD69^+^ CD49a^+^ cells in the subcutaneous model (Fig. 3a).

### E7-TriMix mRNA induces long-lasting memory responses

The rationale behind every therapeutic anticancer vaccine is not only to induce potent antigen-specific effector cells that would allow for a quick eradication of tumour mass but also for the induction of long-lasting memory responses that could prevent disease relapse and metastasis formation^27^. Currently used vaccine adjuvants are known to potently increase the effector function of CD8^+^ T cells, however they have also been shown to promote terminal differentiation of these cells towards the so-called short-lived effector cells, thus significantly reducing the pool of vaccine-induced memory T cells^28^. Therefore, besides the antitumour effect of our mRNA vaccine, we also wanted to evaluate its potential to induce long-lasting memory responses. While CD62L expression is thought to be a characteristic of central memory T cells, the expression of killer cell lectin-like receptor subfamily G1 (KLRG1) and interleukin 7 receptor α-chain (CD127) was shown to be the best indicator of memory/effector fate^29^. For that reason, we assessed the expression of the surface molecules CD62L, CD127 and KLRG1 within the population of E7-DEX^+^ CD8^+^ spleen cells in the E7-TriMix-treated survivors that had rejected genital tract tumours. Due to the very low numbers of cells, we were unable to evaluate the numbers of tissue-residing memory cells in the genital tract tissue. We found that by the end of the contraction phase (day 24 post the last immunization) within the fraction of CD62L^+^ splenocytes there were almost no KLRG1^+^ T cells detected and the majority of events exhibited a CD127^+^ phenotype. Consistently with the previous reports, on day 90, a population of long-lived CD127^+^ KLRG1^+^ cells occurs (Fig. 3b)^29^.

### Tumour location determines the therapeutic efficacy of the vaccine

**Figure 4:**
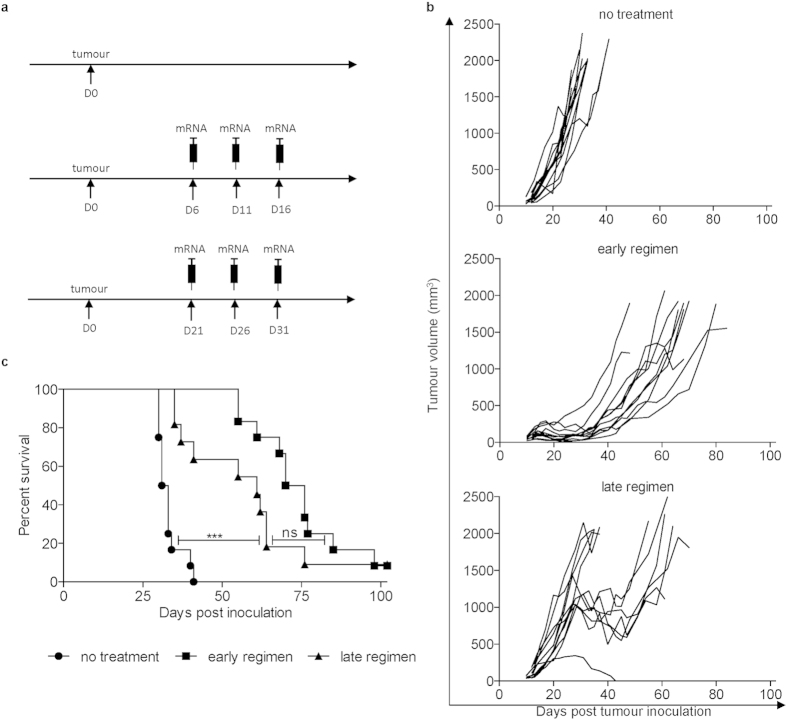
Tumour size determines the efficacy of the therapy in the subcutaneous model. Mice bearing subcutaneous TC-1 tumours were split into three groups as shown in the experimental setting (**a**) and treated as described in Materials and Methods. The tumour growth was monitored 2–3 times a week with a digital calliper. Each line represents an individual mouse (**b**). Survival curves with median survival times: no treatment 32 days, early regimen 73 days, late regimen 61 days (**c**). Data shown are from 2 independent experiments, 11–12 individuals per group. Statistical analysis for (**c**) log-rank test; *** significant at p < 0.001, ns: non significant.

First, we checked whether the induced anti-E7 immune responses could lead to a therapeutic control of subcutaneously implanted TC-1 tumours. For this purpose, two different treatment regimens were tested. For the early vaccination regimen, the mRNA was administered into the tumour-draining subiliac lymph node when the tumours reached an approximate size of 30 mm^3^. For the late vaccination regimen, the therapy was initiated when tumours reached the size of 600 mm^3^. As shown in Fig. 4, a significant delay in tumour growth was achieved for both early and late vaccination regimen.

**Figure 5:**
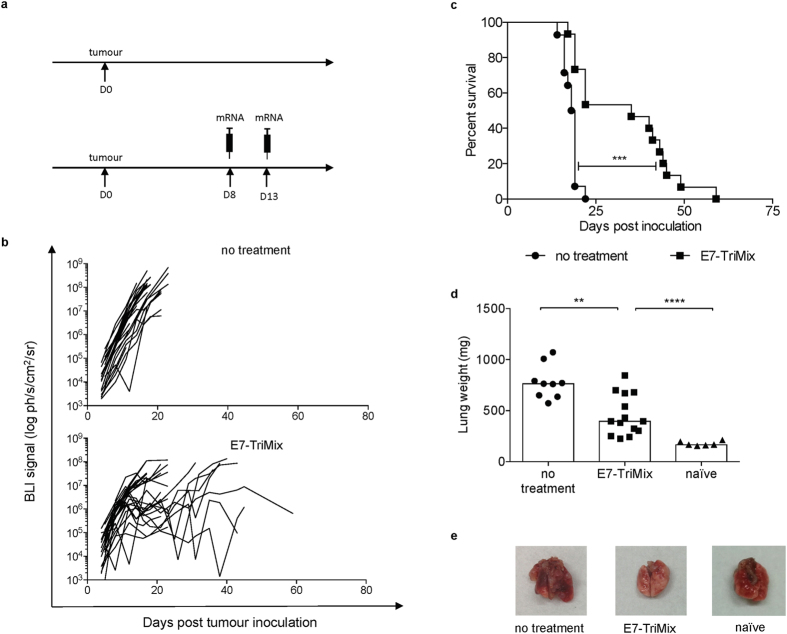
E7-TriMix vaccination controls progression of TC-1 lung tumours. Fluc^+^ TC-1 lung tumour-bearing mice were treated as shown in the experimental setting (**a**). The kinetics of tumour growth were monitored by means of *in vivo* BLI; each line corresponds to an individual mouse (**b**). Survival curves with median survival times: no treatment 18.5 day, E7-TriMix 35 days (**c**). For a randomized group of mice, the lung weight was checked 10 days after the last immunization; each dot represents an individual mouse; the bars represent the median values (**d**). Representative photos of organs from (**d**) are shown (**e**). (**b**,**c**) 2 independent experiments, 22–26 individuals per group; (**d**) 2 independent experiments, 6–14 individuals per group. Statistical analysis for (**c**) log-rank test; *** significant at p < 0.001; for (**d**) Mann-Whitney test; ** significant at p < 0.01, *** significant at p < 0.001.

**Figure 6:**
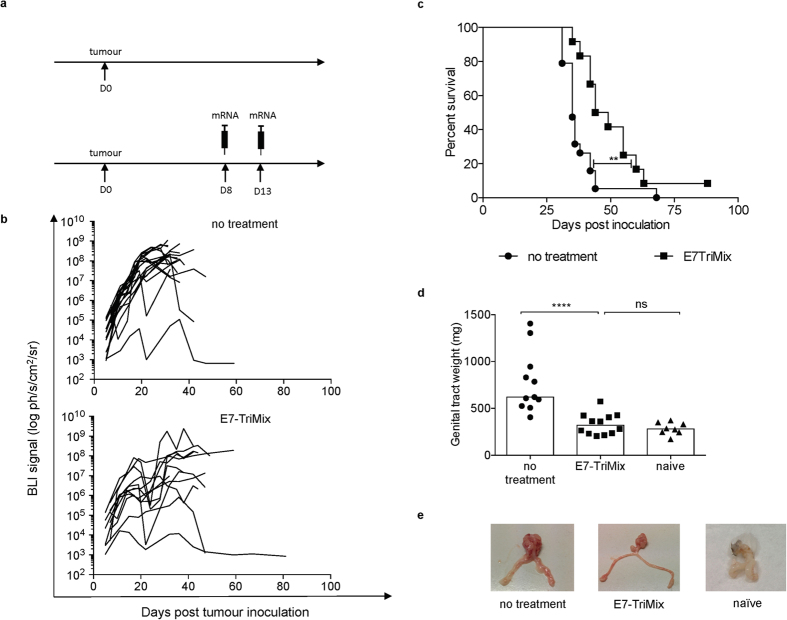
TC-1 tumour lesions located in genital tract are resistant to E7-TriMix treatment. Fluc^+^ TC-1 genital tract tumour-bearing mice were treated as represented in the experimental setting (**a**). The kinetics of tumour growth were monitored by means of *in vivo* BLI; each line corresponds to an individual mouse (**b**). Survival curves with median survival times: no treatment 35 days, E7-TriMix 46.5 day (**c**). The genital tract weight was checked 10 days post the last immunization in a randomized group of mice; each dot represents an individual mouse; the bars represent median values (**d**). Representative pictures of organs from (**d**) are shown in (**e**). (**b**,**c**) 2 independent experiments, 12–19 individuals per group; (**d**) 2 independent experiments, 8–12 individuals per group. Statistical analysis for (**c**) log-rank test; ** significant at p < 0.01; for (**d**) Mann-Whitney test; **** significant at p < 0.0001, ns: non significant.

Considering the rather limited translational value of subcutaneous models in the context of HPV-associated neoplasms, we tested the efficacy of our vaccination also in the orthotopic lung tumour model and the orthotopic genital tract model. This approach is feasible when using TC-1 cells expressing firefly luciferase (TC-1 fluc^+^). Hence, we were able to monitor the growth of orthotopically-implanted tumours using non-invasive *in vivo* bioluminescence imaging (BLI). Although our treatment exerted a potent anticancer effect in the lung tumour model leading to an almost 2-fold increase in the lifetime of treated animals (Fig. 5), its efficacy in the genital tract model was moderate (Fig. 6). The effect of vaccination was also verified by a *post-mortem* weighing of lung or genital tract and confirmed the BLI data (Figs 5d and 6d).

### The immunosuppressive tumour microenvironment limits the therapeutic benefit in the genital tract model

**Figure 7:**
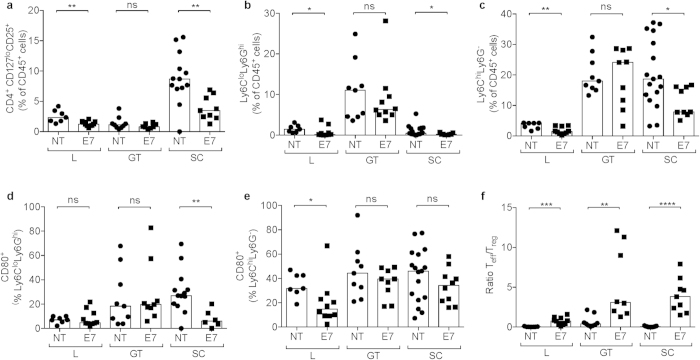
The immunosuppressive tumour microenvironment differs depending on the tumour location. Mice bearing differently located TC-1 tumours were immunized with E7-TriMix as described in Materials and Methods. Ten days post the last immunization (for orthotopic lung and genital tract model) or when tumours reached 1000 mm^3^ (for subcutaneous model), tumour tissue and spleens were isolated and flow cytometric analysis was performed to analyse the numbers of Tregs (**a**), grMDSCs (**b**), moMDSCs (**c**), CD80^+^ grMDSCs (**d**) and CD80^+^ moMDSCs (**e**). (**f**) shows the ratio Teff/Tregs in the different tumour models. Each dot represents an individual mouse; the bars correspond to the median values. Abbreviations: NT: no treatment, E7: E7-TriMix-treated mice, L: lungs, GT: genital tract, SC: subcutaneous tumours. (**a**,**e,f**) 2 independent experiments, 7–13 individuals per group; (**b**–**d**) 2 independent experiments, 7–17 individuals per group. Statistical analysis: Mann-Whitney test; * significant at p < 0.05, ** significant at p < 0.01, *** significant at p < 0.001, **** significant at p < 0.0001, ns: non significant.

The survival experiments revealed that the therapeutic effect of E7-TriMix mRNA was the least pronounced in the genital tract model. To find out the mechanism limiting the efficacy of the treatment in this model, we made a comparative analysis of the TME of lesions located subcutaneously, in the lung or in the genital tract. Since the Teff/Treg ratio has been reported to be an independent prognostic factor for patients with cervical cancer, we first evaluated the numbers of intralesional Tregs (defined as CD45^+^CD3^+^CD4^+^CD8^−^CD25^hi^CD127^lo^) and found that the TME of subcutaneous tumours abounded in Tregs as compared with other tumour locations, although their numbers dramatically dropped upon vaccination^30^. Importantly, the numbers of Tregs in the genital tract tumours were unaffected by the vaccination (Fig. 7a). We then checked the numbers of granulocytic (gr; defined as CD45^+^CD11b^hi^CD11c^−^Ly6C^lo^Ly6G^hi^) and monocytic (mo; defined as CD45^+^CD11b^hi^CD11c^−^Ly6C^hi^Ly6G^−^) myeloid-derived suppressor cells (MDSCs). Here, we found that the genital tract model was the only one that abounded in both MDSC sub-populations. Strikingly, grMDSCs which our group has shown to be more suppressive^31^, constituted around 10% of total immune infiltrate of genital tract tumours whereas in the other two models this value oscillated between 0.3% and 1%. Similarly, the numbers of moMDSC were elevated in genital tract tumours reaching about 20% of tumour immune infiltrate. The numbers of moMDSC significantly decreased upon E7-TriMix treatment in the subcutaneous and the lung tumour model. This phenomenon was, however, not observed in genital tract tumours (Fig. 7b,c). We then verified the numbers of CD80^+^ cells within the MDSC population, since these cells were shown to be more suppressive than their CD80^−^ counterparts^31^,^32^. A prominent contraction of the CD80^+^ population within grMDSCs was observed upon vaccination for subcutaneous tumours. This however again did not apply for genital tract tumours (Fig. 7d,e). The tested models differed also in effector T cell (Teff)/Treg ratios that reach the highest values for the subcutaneous tumours (Fig. 7f).

### Chemoimmunotherapy with cisplatin and E7-TriMix mRNA leads to a complete rejection of genital tract tumours

**Figure 8:**
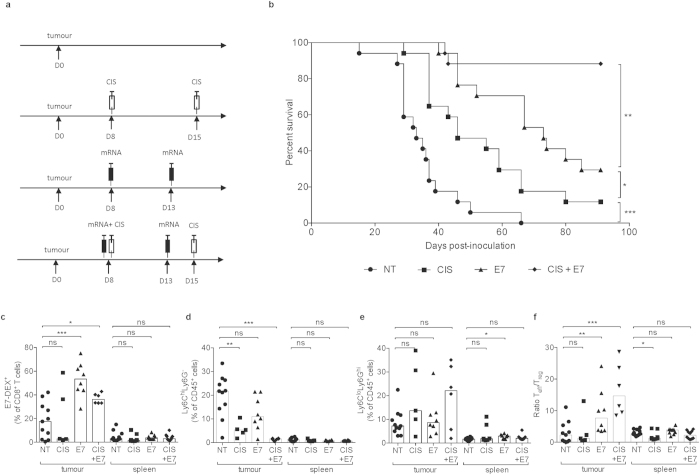
Combination of E7-TriMix mRNA and cisplatin leads to a complete and durable rejection of genital tract tumours. Mice were intravaginally inoculated with fluc^+^ TC-1 tumour cells and treated as represented in the experimental setting (**a**). Survival curves with median survival times: no treatment 35 days, cisplatin 47 days and E7-TriMix 73 days; 88% of mice treated with cisplatin + E7-TriMix survived (**b**). For a randomized group of mice, the tumour microenvironment was checked 10 days after the last immunization: E7-specific T-cell infiltration (**c**), moMDSCs (**d**), grMDSCs (**e**) and ratio Teff/Treg (**f**). Each dot represents an individual mouse, bars correspond to median values. Abbreviations: NT: no treatment, CIS: cisplatin-treated mice, E7: E7-TriMix-treated mice, E7 + CIS: cisplatin and E7-TriMix-treated mice; (**b**) one experiment with 17 individuals per group; (**c**–**f**) 2 independent experiments, 5–11 individuals per group. Statistical analysis for (**b**) log-rank test; * significant at p < 0.05, ** significant at p < 0.01, *** significant at p < 0.001; for (**c**–**f**) Mann-Whitney test; * significant at p < 0.05, ** significant at p < 0.01, *** significant at p < 0.001, ns: non significant.

The E7-TriMix mRNA vaccine reduced the numbers of Tregs in the lung and the subcutaneous tumours, but not in genitally located tumours. Similarly, both MDSC fractions remained high only in genital tract lesions even upon mRNA therapy introduction. We therefore hypothesized that high infiltration with Tregs and MDSCs could be a major culprit of limited therapeutic efficacy of the mRNA vaccine in genital tract model (Fig. 6). Preclinical research revealed the immunomodulating mechanisms of certain chemotherapeutics, resulting in several clinical trials^33^. In this context, cisplatin is being anew acknowledged as a multifunctional anticancer agent. In the recent decade, it became apparent that cisplatin not only inhibits mitosis by its ability to cross-link DNA, but also exerts multiple immunomodulatory effects^34^. Additionally, it has since long been applied in the clinic for the treatment of many types of malignancies, including the HPV-related tumours. For that reason, to solve the problem of unsatisfactory therapeutic outcomes in the genital tract model, we combined the mRNA vaccination with cisplatin treatment. To this aim, genital tract tumour-bearing mice were immunized with E7-TriMix mRNA and treated with cisplatin as described in Materials and Methods. Survival experiments revealed that both cisplatin alone and E7-TriMix mRNA alone prolonged the survival and improved the life quality of the mice (as determined by body weight change, Supplementary Fig. 1), however ultimately most animals succumbed to the tumour. In spite of that, the combination of both treatments led to a complete rejection of neoplastic lesions in 88% of the mice (Fig. 8b). A thorough analysis of the TME revealed a significant drop in the fraction of moMDSCs, despite the fact that the grMDSCs slightly increased (Fig. 8d,e). Moreover, cisplatin treatment reduced the numbers of Tregs, which consequently further elevated the ratio Teffs/Tregs (Fig. 8f). Importantly, a large majority of the above-described relations do not reach statistical significance for spleen cells.

## Discussion

In this study we evaluated the immune responses against the HPV-derived E7 antigen raised after intralymphatic injection of E7-TriMix mRNA in the context of the TC-1 tumour model, inoculated at different anatomical locations. We demonstrated that the immune contexture of the lesions differs depending on tumour location, thus affecting the outcome of the tested therapeutic approach and thereby its translational value. Here, we uncovered that although the vaccine-induced CD8^+^ T cells migrate to the mucosally located tumours, the hostile immunosuppressive TME of these tumours is responsible for limited therapeutic benefit. In the genital tract model, this *status quo* could be associated with the high numbers of tumour-infiltrating MDSCs and Tregs. We show that the immunosuppressive TME within the genital tract can be abrogated by using cisplatin. Thereby, we demonstrate that cisplatin is not only a cytostatic but also an immunomodulatory agent that works synergistically with the E7-TriMix mRNA vaccine, leading to a complete and durable rejection of clinically more relevant orthotopic genital TC-1 tumour lesions.

The expenses of treating HPV-related infections constitute a significant burden for society^35^. Two preventive anti-HPV vaccines were approved under the assumption that eradication of HPV-infection over time would practically rule out the risk of cervical cancer occurrence. However, considering the less than optimal vaccine coverage, there is—apart from preventive measures—still an important need for the development of an efficient therapeutic strategy against HPV-related tumours, particularly against cervical cancer. Multiple therapeutic approaches against cervical cancer have so far been evaluated^7^,^8^. Nevertheless, the promising preclinical data are in sharp contrast with the poor clinical outcomes. The sole exceptions are premalignant lesions, where the therapeutic vaccines led to a full and durable disease regression^36^,^37^,^38^,^39^.

Several obstacles may be lying at the basis of the unsatisfactory translational efficacy of laboratory research in the field of therapeutic vaccines against HPV-derived tumours. First of all, rapidly growing ectopically implanted tumours are most of the time used for research purposes to facilitate these studies. Additionally, very often the treatment is introduced at the moment of tumour-cell inoculation or when the tumour nodules are palpable. As rightly recalled by the group of Hans Schreiber, such approach can in fact target inflammatory swelling and a few viable tumour cells and is therefore not applicable to tumours of clinically detectable size^40^,^41^,^42^,^43^. A recently published systematic analysis of experimental immunotherapies revealed that a large majority of reported immunotherapies target tumours of an average size of only 45 mm^3^ with median time of treatment initiation at 5 days post inoculation^44^. Inspired by these facts, we decided to test our vaccination approach in two variants of ectopic tumours (palpable versus long-established tumours). Surprisingly, the E7-TriMix mRNA vaccine still exerted a potent therapeutic effect on the long-established tumours that were twenty times larger in size than the palpable tumours (Fig. 4). Nevertheless, the use of scarcely relevant subcutaneous tumours to test the efficacy of immunotherapeutic approaches for cervical cancer in many cases brings the risk of over-optimistic data. Although, the long-established subcutaneous tumours mimic more closely the situation in the clinic (patients are diagnosed with already long-existing neoplastic lesions), they still ignore the role of the local environment. To fill this gap, we chose to look at orthotopic tumour locations.

Given the complexity of T-cell homing pathways into the mucosal tissue, it has also been proposed that in order to increase immune responses against mucosally located tumours, the vaccine should be administered at mucosal sites^12^,^13^,^45^. Indeed, mucosal vaccines have since long been considered as a very promising approach, particularly due to the ease of administration (*e.g.* intranasal) and the presence of the so-called mucosal phenotype has been associated with strong anti-tumour protection^24^,^25^,^46^. In contrast to this hypothesis, we found that intralymphatically administered E7-TriMix vaccine exerted a therapeutic control on both lung lesions and genital tract lesions (Figs 5 and 6), indicating that the vaccine-induced T cells can concentrate at the place where the antigen reservoir is available, even though they lacked the CD103 and CD49a expression and the vaccine was not given within the mucosal compartment^21^. In the same vein, some other groups report that—although CD103 is strongly indicative of tissue residence—it should be cautiously used as a definitive marker of Trm cells, since in many organs these cells lack CD103 expression^47^,^48^. The fact that TC-1 tumour cells do not express E-cadherin, a natural ligand for CD103, confirms these findings^13^. Therefore, in the context of murine model of cervical cancer, CD103 seems to be dispensable for T-cell retention within the tumour tissue while CD69 expression—since its upregulated upon TCR engagement—might rather be indicative of an ongoing inflammatory process/presence of antigen^49^. A particularly interesting observation has recently been made by Iwasaki’s group that described the flow of submucosal DCs from vaginal mucosa to the inguinal lymph nodes, indicating that these lymph nodes play a critical role in the induction of immune responses against pathogens that invade the host through genital tract^50^. Since DCs from all mucosal tissues have to migrate to the nearest lymph node in order to prime naïve T cells, we believe that with our approach we omit the challenging step of mucosal delivery of mRNA vaccine by directly targeting the place where the immune responses arise^51^,^52^. Our group proposed and assessed the feasibility of using direct intralymphatic injection of mRNA in order to circumvent the use of patient-specific DCs for vaccination. We clearly showed that intralymphatic mRNA injection is easy, safe and at least as potent as DC vaccination^20^. Several clinical trials in which mRNA is administered intralymphatically (*i.e.* intranodally) are now ongoing (EudraCT2012-005572-34, NCT01684241, NCT02035956).

Seeing that the efficacy of the vaccine in the genital tract model was more limited than that observed for lung tumours (Figs 5 and 6), we hypothesized that the therapeutic effect of the mRNA-based immunotherapy in the context of TC-1 lesions can be affected by immunosuppressive organ-specific microenvironment. Indeed, we found that the genital tract tumours were abundantly infiltrated by MDSCs when compared with subcutaneous tumours and lung tumours (Fig. 7b,c). Importantly, the numbers of both grMDSCs and moMDSCs remained unaffected by the vaccination in genital tract lesions, whereas being significantly reduced upon treatment of lung tumours and subcutaneous tumours. A characteristic feature of myeloid cells is their plasticity in response to tumour-derived stimuli. Consequently, these cells often share not only phenotypic but also functional features^53^,^54^. Therefore, although the markers used in our study allow for a safe distinction of grMDSC, a population of moMDSC is likely to comprise also other cell types of similar phenotype, such as DC-like macrophages or inflammatory macrophages, as elegantly depicted by van der Sluis *et al*.^53^ Unfortunately, neither in human nor in mice, the current state of knowledge does allow for a definitive distinction of myeloid subpopulations infiltrating tumour tissues without testing their suppressive functions. Similarly to the MDSCs, the numbers of Tregs were reduced upon treatment of lung and subcutaneous tumours, but not of genital tract tumours (Fig. 7a). Interestingly, the high Treg infiltration of genital TC-1 lesions was also reported by Nardelli-Haefliger and colleagues^14^. These TME dissimilarities within genetically the same, but differently located tumour cell line prove that the dynamic interplay between tumour cells and their environment cannot be neglected.

Nowadays, it has become evident that a combination therapy where standard treatments, such as chemotherapy, meet immunotherapy could be beneficial for patients^55^,^56^. Here, we demonstrate that combination of mRNA vaccination with cisplatin treatment leads to a complete tumour eradication in a clinically more relevant orthotopic model of cervical cancer (Fig. 8). Additionally, we evaluated the effects of this combination therapy on the composition of the TME. In first instance, we checked the numbers of tumour-infiltrating T cells. Whereas several groups demonstrated increased numbers of effector cells upon treatment with cisplatin^56^,^57^, this phenomenon was not observed in our experiments nor in the study reported by van der Sluis and colleagues^58^. It is plausible that the tumour burden had been completely rejected by the day of analysis, as seen on immunofluorescent sections (Fig. 1c). Consequently, as the antigen reservoir was absent, the E7-specific T cells might have entered a contraction phase. Several mechanisms have been proposed on how cisplatin regulates lytic capacities of the effector cells. For instance, it has been suggested that cisplatin can increase Fas-expression on tumour cells and as such make them more vulnerable to effector antigen-specific T cells^59^. It has also been proposed that cisplatin pretreatment increases the permeability of tumour cells to granzyme B secreted by effector cells^60^. More recently, van der Sluis *et al*. demonstrated that cisplatin renders the tumour cells more susceptible to TNF-α secreted by effector cells^58^. In the context of the TC-1 cell line, tumour cells were shown to upregulate MHC I upon *in vitro* treatment with cisplatin^56^. In this study, in concert with previous findings, we demonstrated that cisplatin treatment decreases the frequency of tumour-infiltrating immunosuppressive cells (Fig. 8d,e)^57^,^61^,^62^.

An important finding of our study is the fact that the lion’s share of vaccine-induced antigen-specific T cells was observed in the tumour nest and not in the spleen, indicating that—in the context of TC-1 tumour model—the numbers of antigen-specific T cells detected in the blood are not fully indicative of the ongoing immune responses and thus underestimating the vaccine efficacy (Figs 1a,b and 8c–f). This raises a question about the reliability of currently applied techniques for immunomonitoring of cancer patients. The prognostic value of immune responses detected in blood samples of CIN2/3 patients has already been questioned by Maldonado *et al*.^63^ Similarly, although Bagarazzi *et al*. claim that no significant correlation exists between Tregs and the potency of E7-specific immune responses, it is important to highlight that the authors evaluated peripheral blood Tregs, which might not be fully representative for the Tregs in the tumour nest^64^. In the context of preclinical cervical cancer models, particularly in the TC-1 model, only very few studies look at the tumour-resident immunosuppressive cells, while it has been shown that *e.g.* tumour-derived MDSCs possess stronger suppressive capacities than their non-infiltrating homologues^65^. Here, we provide a direct multi-parameter preclinical evidence for the superiority of tumour sampling for the monitoring of anticancer immunity in the context of TC-1 tumour model. We demonstrate that all tested parameters (*i.e.* infiltration of antigen-specific T cells, infiltration of immunosuppressive cells and IFN-γ secretion) were much more pronounced in the tumour samples, whereas the spleen samples gave results on the border of statistical significance, often comparable with non-treated individuals, even when complete tumour regression was seen (Fig. 8).

Our data demonstrate that, the intralymphatically administered E7-TriMix vaccine induces antigen-specific T cells that are attracted to and retain within the tumour tissue despite the fact that they do not express mucosa-associated markers such as CD103 and CD49a. Although in large numbers, these cells can be insufficient to cease the tumour progression^66^. Based on the side-by-side comparison of the ectopic and the orthotopic tumours, we postulate that it is of crucial importance to identify the tissue-specific signatures of tumour lesions in order to design appropriate multi-target therapies and maximize the antitumour immune responses. We therefore supplemented our mRNA vaccination approach with cisplatin treatment. This combination therapy granted the possibility to act specifically on the hostile tumour environment of genital tract tumours and as a consequence rendered the mice tumour-free. Our study also highlights the need for the evaluation of the immunological status of the tumour to ensure the translational value of preclinical research, since we report here that significant changes can only be caught in the tumour tissue, not in the periphery. We hope that this finding will contribute to further improvement in cancer patient monitoring, particularly in the context of cervical cancer where tumour lesions are directly accessible.

## Methods

### Animals

All experiments were conducted on 6 to 12 weeks old C57BL/6 female mice purchased from Charles River Laboratories (France). The animals were maintained and treated in accordance to the institutional and European Union guidelines for animal experimentation. The animals had *ad libitum* access to food and water. All *in vivo* animal studies were approved by the Ethical Committee of the Vrije Universiteit Brussel and the data collection was stopped when one of the humane endpoints dictated by Ethical Committee of Vrije Universiteit Brussel was met. In this regard mice were sacrificed when tumour size exceeded 2000 mm^3^ or the body weight loss was greater than 20%.

### Cell lines

The fluc^+^ TC-1 cell line was kindly provided by prof. Denise Nardelli-Haefliger (Centre Hospitalier Universitaire Vaudois and University of Lausanne, Lausanne, Switzerland). These cells were modified to stably express HPV16-E6 and HPV16-E7 proteins as described elsewhere^67^. The expression of the viral proteins was confirmed by RT-PCR. Tumour cells were cultured in RPMI 1640 supplemented with 10% foetal calf serum, 100 IU penicillin, 100 μg/ml streptomycin, 2 mM L-glutamine, 1 mM sodium pyruvate, non-essential amino acids and 0.4 mg/mL G418. Prior to inoculation, the expression of E7 and firefly luciferase by tumour cells was checked and a Mycoplasma test was performed (PCR).

### Randomization and blinding

Upon arrival, the mice were given marks on the ears or tail and were randomly ascribed to individually ventilated cages in which they were housed until the end of experiment, regardless of the experimental group to which they were ascribed upon randomization. This allowed blinded measurements of the tumour growth. Immunofluorescence data was collected and analysed by a blinded pathologist. For all experiments mice were randomized for tumour burden based on *in vivo* BLI or calliper measurements. The animals were ascribed to various experimental groups in a tumour size-matched or luminescence signal-matched manner.

### Sample size and replicates

The effect of the therapy was unknown, no power calculations were therefore performed. The number of animals and replicates are stated in the figure legends. No outliers were excluded.

### Experimental procedures

#### Subcutaneous tumours

Mice were inoculated subcutaneously with 20.000 fluc^+^ TC-1 tumour cells (in 50 μl PBS) in the right flank. The E7-TriMix mRNA vaccine was administered intralymphatically into the right subiliac lymph node when the tumours reached an approximate size of 30 mm^3^ (for early vaccination regimen, day 6) or 600 mm^3^ (for late vaccination regimen, day 21). Animals were immunized three times with 5-day intervals.

#### Orthotopic lung and genital tract model

For the lung model, mice were inoculated with 10.000 fluc^+^ TC-1 cells (in 20 μl PBS) by means of intercostal injection. For the genital tract model, mice were firstly given hormonal treatment with DepoPromone (3 mg/mouse) on day 0. On day 3, mucosa of genital tract was destroyed by intravaginal administration of 4% Nonoxyl–9. On day 4 genital tract was washed with PBS and 15.000 fluc^+^ TC-1 cells (in 20 μl PBS) were instilled intravaginally. On day 7, animals were randomized based on *in vivo* BLI data and on day 8 immunized into both left and right subiliac lymph nodes. The immunization was repeated 5 days later. The tumour growth and body weight were monitored every 2–3 days. Ten days after the last immunization, a randomized group of mice was sacrificed for immune response monitoring (IFN-γ ELISPOT, flow cytometry).

#### Combination therapy with E7-TriMix mRNA and cisplatin

Combination therapy was performed in the genital tract tumour model. Mice were inoculated with tumour cells and randomized as described before. On day 8, mice received E7-TriMix vaccination (two times with 5-day interval) with or without cisplatin given in four doses (4 mg/kg) on days 8, 9, 15 and 16.

### Intralymphatic immunization

Mice were anesthetized using a solution of Ketamine (Ceva) and Rompun (Bayer) diluted in sterile PBS, administered intraperitoneally. The mRNA was injected into the lymph nodes using a BDMicro-Fine + syringe (0.3 mm × 8 mm). For each injection 2.5 μg of each of the TriMix components and 5 μg for E7 antigen was used.

### *In vivo* Bioluminescence Imaging (BLI)

Isoflurane 2% was applied as a gas anaesthetic. D-luciferin (Promega, The Netherlands) was administered intraperitoneally in a volume of 100 μl (30 mg/ml) per 20 g body weight. After 10 minutes, the fluorescence was measured by the BioSpace detection system. The acquisition time was equal to 4 minutes. Obtained data were analysed by use of M3Vision software.

### Flow cytometry

For surface molecules, the cells were washed twice with PBS/BSA/Azide and were incubated with a desired antibody mixture for 30 minutes at 4 °C. The excess of antibodies was washed out prior to the read-out. The E7_(RAHYNIVTF)_-dextramer staining was performed according to the manufacturer’s instructions (Immudex, Denmark). Data was acquired on an LSRFortessa cytometer and analysed with BDFACSDiva Software, version 6.2 (Becton Dickinson). The antibodies used for flow cytometry are listed in Table 1.

### Plasmids and messenger RNA

The formulation of TriMix components was described previously^20^. The sequence encoding the HPV16-E7 protein was cloned in-frame between the signal sequence and the transmembrane and cytoplasmic regions of human DC-LAMP. This chimeric gene was cloned in the peTheRNA plasmid that was enriched with *i.a.* a translation enhancer at the 5′ end and an RNA stabilizing sequence at the 3′ end. Prior to injection, mRNA was precipitated with LiCl, purified with 70% ethanol, dissolved in water and Hartmann Solution (ratio 1:4) and left at room temperature for 30 minutes. The volume administered into a single lymph node was always equal to 10 μl. mRNA quality was monitored by capillary gel electrophoresis (Agilent, Belgium).

### Peptides

E7-HPV16 (49–57) peptide was purchased from Eurogentec, Belgium. The peptides were used at a final concentration of 2.5 μg/ml.

### *Ex vivo* sample preparation

After removal, spleens were mashed through a sterile 40-μm cell strainer using a plunger of a syringe and red blood cells were lysed. Where indicated, a MACS-purified CD8^+^ T cell population was used (Miltenyi Biotec, Leiden, The Netherlands). After removal, tumour tissues were homogenized mechanically—without enzymatic digestion—using sterile glass tubes and sterile glass plungers. The population of tumour-infiltrating immune cells was enriched by density centrifugation using Lympholyte®-M Cell Separation Medium (Cedarlane, USA) according to the manufacturer’s instructions.

### IFN-γ ELISPOT

In all experiments a murine IFN-γ ELISpot kit (Diaclone, USA) was used. Microtiter plates (96-well, Diaclone, USA) were coated overnight with anti-IFN-γ capture antibody according to the manufacturer’s instructions and aspecific binding was blocked with use of TC-1 cell line culture medium for 2 h at 37 °C. When working with splenocytes, the CD8^+^ population was firstly enriched using MACS separation kit Immune cells derived from tumours were seeded into microtiter plates without enrichment for the CD8^+^ population. 2*10^5^ cells per well were incubated with or without E7 peptides for 36 h. The spots were developed according to the manufacturer’s instructions. As a positive control, T cells were stimulated with anti-CD3/anti-CD28 beads. Spots were counted using an AID ELISpot reader (Autoimmun Diagnostika GmbH, Strassberg, Germany). The spots forming unit (SFU) values were normalized, based on the percentage of CD8^+^ T cells detected in the cell suspension by flow cytometry.

### Immunofluorescence analysis

Immunofluorescence was done on 7 μm cryosections. Briefly, after thawing sections were blocked with 10% normal donkey serum for 1 h, followed by an overnight incubation at 4 °C with primary antibodies: rabbit anti-mouse Ki67 (Acris Antibodies GmbH, Germany) and rat anti-mouse CD8 (Abcam, UK). After a PBS wash, sections were incubated with secondary anti-rat and anti-rabbit antibodies for 1 h followed by a PBS wash and mounting with DAPI. Pictures were taken with a Pathway435 microscope using Attovision/IPLab software.

### Statistics

Statistical analyses were performed using GraphPad Prism 6 for MacOSX. Analyses were performed with unpaired two-tailed Mann-Whitney test or log-rank test in case of Kaplan-Meier curves.

## Additional Information

**How to cite this article**: Bialkowski, L. *et al*. Intralymphatic mRNA vaccine induces CD8 T-cell responses that inhibit the growth of mucosally located tumours. *Sci. Rep.* **6**, 22509; doi: 10.1038/srep22509 (2016).

## Supplementary Material

Supplementary Information

## Figures and Tables

**Table 1:** The table lists all the antibodies used for flow cytometric analyses.

Antibody	Clone	Fluorochrome	Company
CD3	17A2	PE-Cy7	BD
CD4	RM4-5	AlexaFluor700	BD
CD8	53-6.7	APC-H7	BD
PD-1	RMP1–30	PE	BioLegend
KLRG-1	2F1/KLRG1	APC	BioLegend
KLRG-1	2F1/KLRG1	PE	BioLegend
CD127	SB/199	PerCPCy5.5	BioLegend
CD127	SB/199	PE-CF594	BD
CD62L	MEL-14	PE-Cy7	BioLegend
CD103	M290	PerCPCy5.5	BD
CD49a	HMa1	PE	BioLegend
CD69	H1.2F3	Biotinilated	BD
CD45	30-F11	V450	BD
CD11c	HL3	PerCPCy5.5	BD
CD11b	M1/70	PE	BioLegend
Ly6C	AL-21	PECy7	BD
Ly6G	1A8	AF647	BioLegend
CD80	Biotinylated, in-house prepared from hybridoma 16-10A1		
E7 Dextramers	n/a	APC	Immudex
