# Supplementary material for: Intralymphatic mRNA vaccine induces CD8 T-cell responses that inhibit the growth of mucosally located tumours
Source: Sci Rep. 2016 Mar 2;6:22509. doi: 10.1038/srep22509 (PMC4773884; doi:10.1038/srep22509)
Supplement: Supplementary Information [file srep22509-s1.pdf]

Title: Intralymphatic mRNA vaccine induces CD8 T-cell responses that inhibit the growth of mucosally located tumours

Lukasz Bialkowski[1], Alexia van Weijnen[1], Kevin Van der Jeught[1], Dries Renmans[1], Lidia Daszkiewicz[1], Carlo Heirman[1], Geert Stangé[2], Karine Breckpot[1], Joeri L. Aerts[1,+], and Kris Thielemans[1,+,*]

[1] Laboratory of Molecular and Cellular Therapy, Vrije Universiteit Brussel, Laarbeeklaan 103E, 1090 Brussels, Belgium.

[2] Diabetes Research Center, Vrije Universiteit Brussel, Laarbeeklaan 103E, 1090 Brussels, Belgium.

[+] These authors share senior authorship

[*] Corresponding author: Kris Thielemans, Laboratory of Molecular and Cellular Therapy, Vrije Universiteit Brussel, Laarbeeklaan 103E, 1090 Brussels, Belgium, tel. (+32) 02 477 45 69, e-mail: Kris.Thielemans@vub.ac.be

Supplementary Information

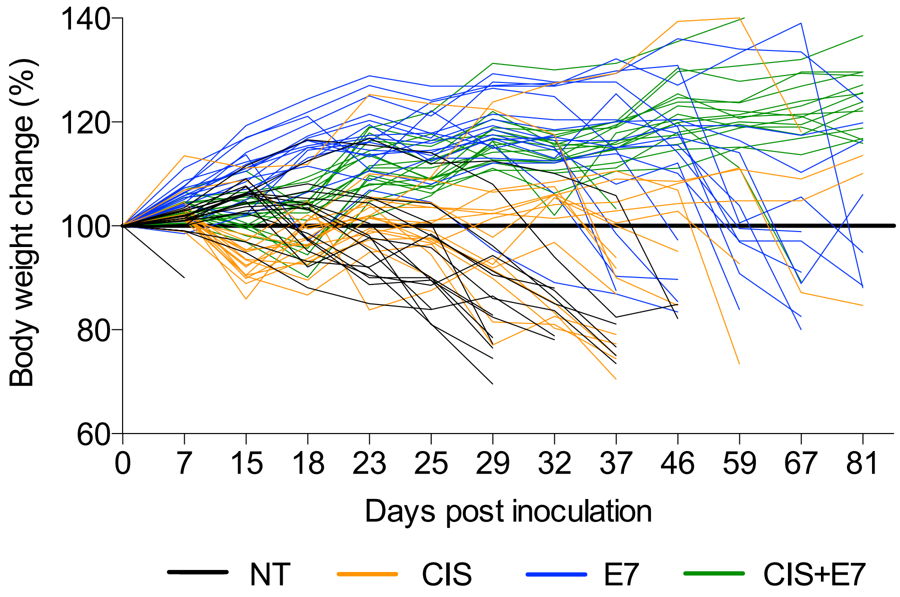

Supplementary Figure 1. Cisplatin treatment together with E7-TriMix immunization exerts a potent effect on the life quality of treated mice.

Mice bearing genital tract tumors were treated according to the experimental setting described in Fig. 8. Body weight was monitored 1-2 times a week in order to evaluate the life quality of treated animals. Each line represents an individual mouse; the value of 100% indicates the body weight on the day of tumor inoculation. Data shown are based on one experiment with 17 individuals per group. Abbreviations: NT: no treatment, CIS: cisplatin-treated mice, E7: E7-TriMix-treated mice, CIS+E7: cisplatin and E7-TriMix-treated mice.
